# Baron Larrey

**Published:** 1864-04

**Authors:** 


*Baron Larrey.*

It must be a source of pride to the profession of military surgery in France, that it has been so honored in the person of one of its chiefs. Public statues and public honors have been freely accorded to him. "The name of Larrey is inscribed on the Arc de Triomphe amidst the names of the captains who, under the Republic, saved France; who, under the Empire, made it so great and powerful. Napoleon was inclined to do still more for his surgeon-in-chief when he said, 'What a man, what a brave and worthy man, is Larrey! What care was given by him to the army in Egypt, in the passage of the desert, after Saint Jean de Acre, and in Europe! I conceived for him an esteem which was never shaken for an instant. If ever the army should raise a column to Gratitude, it ought to erect it to Larrey.'"

What a strange series of events have now led the son of the Larrey whose memoirs we have been glancing at, to be to the second Empire what his father was to the first! A Barron Larrey is still the personal and confidential surgeon of the Emperor Napoleon, his surgeon-in-chief in the fields of an Italian campaign; and as a member of the Council of Health of Armies in the Ministry of War, and as President of the Imperial Academy of Medicine, still does honor to the name by placing it amongst the most conspicuous in the roll of military surgeons in France.—*London Lancet, March*, 1864.

